# Real-world treatment persistence in patients with rheumatoid arthritis initiating DMARDs in Germany—a health insurance claims data analysis

**DOI:** 10.1007/s00393-023-01323-8

**Published:** 2023-02-09

**Authors:** Christoph Fiehn, Silke Zinke, Jennifer S. Haas, Dominic Meise, Julia Theil, Margot Gurrath, Hans-Dieter Orzechowski

**Affiliations:** 1 Rheumatology and Clinical Immunology Baden-Baden, Beethovenstr. 2, 76530 Baden-Baden, Germany; 2 Rheuma Praxis Zinke, Berlin, Germany; 3 Xcenda GmbH, Hanover, Germany; 4 Galapagos Biopharma Germany GmbH, Munich, Germany

**Keywords:** Antirheumatic agents, Biological therapy, Rheumatoid arthritis, Therapeutics, Treatment patterns, Antirheumatika, Biologische Therapie, Rheumatoide Arthritis, Therapeutika, Behandlungsmuster

## Abstract

**Objective:**

To investigate treatment patterns in patients with rheumatoid arthritis (RA) in Germany who had previously received conventional synthetic (cs) or biologic (b) disease-modifying antirheumatic drugs (DMARDs).

**Methods:**

Patients with RA who initiated treatment with a csDMARD, bDMARD, or Janus kinase (JAK) inhibitor between 2017 and 2018 and who had previously received csDMARD or bDMARD therapy were retrospectively selected from the Institute for Applied Health Research Berlin GmbH (InGef). Time on treatment and discontinuation risk were assessed using the Kaplan–Meier method. Cox regression identified variables associated with an increased discontinuation risk.

**Results:**

A total of 990 patients had received prior csDMARD therapy; 375 had received prior bDMARD therapy. Tumor necrosis factor (TNF)-α inhibitors and JAK inhibitors were the most commonly prescribed DMARD class in those previously treated with a csDMARD or bDMARD, respectively. In both cohorts, more patients received DMARD monotherapy than combination therapy. In the prior csDMARD cohort, median time on treatment was 276, 252, and 148 days with JAK inhibitors, TNF‑α inhibitors, and csDMARDs, respectively, and those treated with JAK or TNF‑α inhibitors were less likely to discontinue treatment than those on csDMARDs (log-rank test *p*-value < 0.01 for both comparisons); no significant differences were found within the prior bDMARD cohort.

**Conclusion:**

This is among the first detailed analyses of RA treatment patterns in a real-world setting in Germany since the introduction of JAK inhibitors. TNF‑α inhibitors were the most commonly prescribed DMARD after failure of an initial csDMARD, while JAK inhibitors were the most common among patients previously treated with a bDMARD. In both groups, monotherapy with bDMARD or targeted synthetic DMARD was common. In the prior csDMARD cohort, treatment duration was significantly longer with JAK or TNF‑α inhibitors than with csDMARDs.

**Supplementary Information:**

The online version of this article (10.1007/s00393-023-01323-8) includes the tables S1–S4 and figures S1–S2.

## Abstract (de)

**Zielsetzung:**

Untersuchung der Behandlungsmuster von Patienten mit rheumatoider Arthritis (RA) in Deutschland mit vorheriger Behandlung mit konventionellen synthetischen (cs) oder biologischen (b) krankheitsmodifizierenden Antirheumatika (DMARD).

**Methoden:**

Patienten mit RA, die zwischen 2017 und 2018 eine Therapie mit csDMARD, bDMARD oder einem Januskinase(JAK-)Inhibitor begonnen haben und mit csDMARD oder bDMARD vorbehandelt waren, wurden retrospektiv aus der Forschungsdatenbank des Instituts für angewandte Gesundheitsforschung Berlin (InGef) ausgewählt. Die Behandlungsdauer und das Abbruchrisiko wurden mit der Kaplan-Meier-Methode ermittelt. Parameter für ein erhöhtes Abbruchrisiko wurden über eine Cox-Regressionsanalyse identifiziert.

**Ergebnisse:**

Insgesamt waren 990 Patienten mit csDMARD bzw 375 mit bDMARD vorbehandelt. Tumornekrosefaktor(TNF-)α-Inhibitoren bei den mit csDMARD vorbehandelten und JAK-Inhibitoren bei den mit bDMARD vorbehandelten Patienten waren die am häufigsten verordneten Therapien. In beiden Kohorten wurden mehr DMARD-Monotherapien als Kombinationstherapien eingesetzt. Die mediane Behandlungsdauer bei mit csDMARD vorbehandelten Patienten lag bei 276 (JAK-Inhibitoren), 252 (TNF-α-Inhibitoren) bzw. 148 Tagen (csDMARD). Bei Therapie mit JAK- oder TNF-α-Inhibitoren kam es seltener zu Therapieabbrüchen als mit csDMARD (Log-Rank-Test: *p*-Wert < 0,01 für beide Vergleiche). Die Kohorte der mit bDMARD vorbehandelten Patienten zeigte keine signifikanten Unterschiede.

**Schlussfolgerungen:**

Dies ist eine der ersten Analysen zum Behandlungsmuster der RA unter realen Bedingungen in Deutschland seit der Einführung von JAK-Inhibitoren. Nach Vorbehandlung mit csDMARD wurden v. a. TNF-α-Inhibitoren, bei mit bDMARD vorbehandelten Patienten überwiegend JAK-Inhibitoren verschrieben. Bei beiden Gruppen waren Monotherapien mit bDMARD oder tsDMARD häufig. In der mit csDMARD vorbehandelten Kohorte war die Behandlungsdauer signifikant länger mit JAK- oder TNF-α-Inhibitoren als mit csDMARD.

## Introduction

The German Society for Rheumatology guidelines for the treatment of rheumatoid arthritis (RA) advocate a treat-to-target approach to achieve sustained remission or, in established disease, at least low disease activity [1]. The guidelines recommend initial treatment with a conventional synthetic (cs) disease-modifying antirheumatic drug (DMARD), preferably methotrexate (MTX) [1]. If a patient has an inadequate response to initial csDMARD therapy, the recommended treatment is a combination of csDMARDs, or if unfavorable prognostic factors are present, a combination of a csDMARD with a biologic (b) or targeted synthetic (ts) DMARD [1]. In addition to efficacy, factors such as safety, costs, and patient perceptions are important considerations in RA management [2, 3].

Many studies of RA treatment patterns in Germany report data collected from patients treated before the approval of Janus kinase (JAK) inhibitors in 2017 [4–6]. Our aim was to investigate baseline characteristics and therapeutic pathways in patients with RA treated with DMARDs in Germany following the introduction of JAK inhibitors, with a specific focus on those who had received prior csDMARD or bDMARD therapy.

## Methods

### Study design

The analysis was performed retrospectively using anonymized German health insurance data from the Institute for Applied Health Research Berlin GmbH (InGef) research database (formerly known as the German Health Risk Institute Database) [7]. The database includes claims data of approximately 60 different statutory health insurances covering over 4 million patients who were distributed across Germany and who were already adjusted for age and sex with respect to the overall German population (as per Federal Office of Statistics [DESTATIS, 8]). Data protection regulations in Germany were fully adhered to.

### Study population

**Fig. 1:**
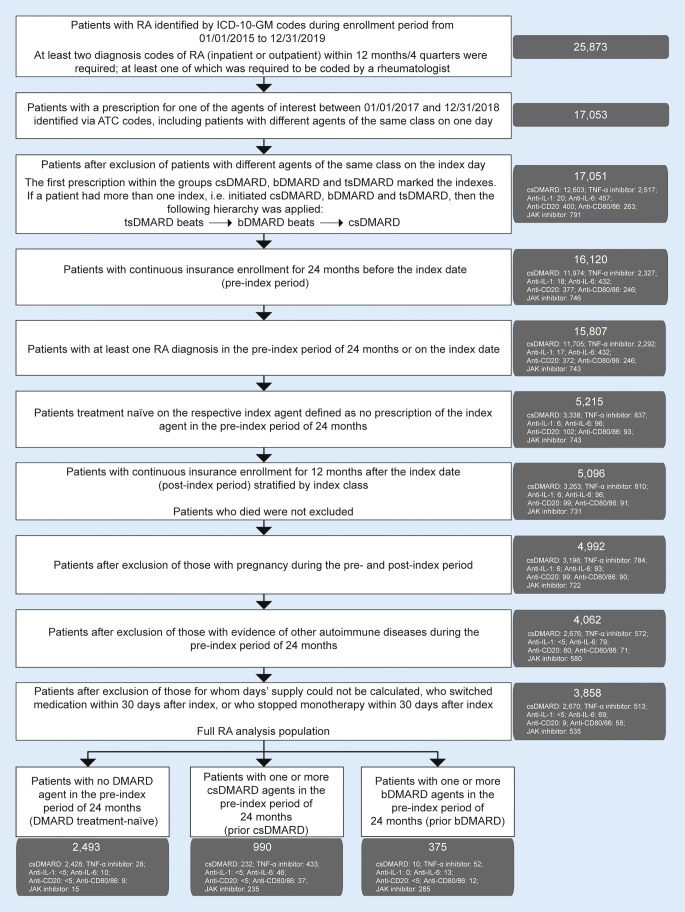
Patient flowchart. *ATC* Anatomical Therapeutic Chemical, *bDMARD* biologic disease-modifying antirheumatic drug, *CD* cluster of differentiation, *csDMARD* conventional synthetic DMARD, *ICD-10-GM* International Statistical Classification of Diseases and Related Health Problems, 10th revision, German Modification, *IL* interleukin, *JAK* Janus kinase, *RA* rheumatoid arthritis, *TNF* tumor necrosis factor, *tsDMARD* targeted synthetic DMARD

Patients with RA were identified by applying the International Statistical Classification of Diseases and Related Health Problems, 10th revision, German Modification (ICD-10-GM) codes (Table S1). Patients were required to have had at least two RA diagnoses within 12 months (i.e., four quarters), with at least one of them coded by a certified rheumatologist (*Innere Medizin/Rheumatologie*, see Table S1). Patients with RA who were treated with a csDMARD, bDMARD, or tsDMARD (JAK inhibitor) between 01 January 2017 and 31 December 2018 were selected (Fig. S1; Table S2). Initiation of treatment with a respective csDMARD, bDMARD, or JAK inhibitor was considered the index event. Patients had a minimum 24-month pre-index period and a minimum 12-month post-index period. If a patient had more than one index event, the following hierarchy was applied: JAK inhibitor > bDMARD > csDMARD. Overall, individuals were required to have a 24-month period free from the respective index agent. A patient flowchart is shown in Fig. 1.

Patients were stratified according to the index event: csDMARD (MTX, leflunomide [LEF], sulfasalazine [SSZ], hydroxychloroquine [HCQ]), bDMARD (tumor necrosis factor [TNF]-α inhibitor, anti-interleukin [IL]-1, anti-IL‑6, anti-cluster of differentiation [CD]20, or anti-CD80/86 agent), or tsDMARD (JAK inhibitor; at the time of the analysis, tofacitinib and baricitinib were the only JAK inhibitors available in Germany). The identified individuals were also stratified by therapy type (monotherapy or combination therapy). Patients initiating monotherapy were required to remain on monotherapy for at least 30 days. For combination therapy, patients who received csDMARD prescriptions within 45 days prior to and/or 45 days after the index date were observed; only combinations with a csDMARD (e.g., bDMARD plus csDMARD) were considered valid. Hence, patients treated with implausible combinations, such as a bDMARD plus bDMARD/tsDMARD at any time between 01 January 2017 and 31 December 2018 were excluded from the analysis.

The current analysis focused on patients who had been prescribed at least one previous csDMARD or bDMARD in the 24-month pre-index period (i.e., patients who were not DMARD naïve when treatment with a csDMARD, bDMARD, or JAK inhibitor was initiated as the index event). These patients will hereafter be referred to as the prior csDMARD and prior bDMARD cohorts, respectively.

Patients were excluded if pregnancy occurred in the pre- or post-index period, or if autoimmune diseases other than RA (ankylosing spondylitis, Crohn’s disease, hidradenitis suppurativa, juvenile idiopathic arthritis, lupus, non-infectious uveitis, psoriasis, psoriatic arthritis, ulcerative colitis) were diagnosed (based on ICD-10-GM codes; Table S3) in the 24-month pre-index period or in the index quarter.

### Study measures

Treatment patterns were analyzed in the 12-month post-index period and were assessed in terms of treatment duration, persistence, treatment discontinuation, and treatment switches. Time on treatment was defined as the day of treatment initiation until discontinuation (the last day of medication supply, which was based on the date that the prescription was dispensed and medication pack size in combination with dosage recommendations as per the package leaflet), switch (prescription of a different drug class), cycling (starting treatment with a new drug within the same class), or augmentation of a new drug (≥ 30-day overlap in the supply of ≥ 2 index drugs without discontinuation of the previous treatment). Persistence was defined as an absence of treatment interruption of > 60 days in the follow-up period. We also assessed prior DMARD treatments in the 24-month pre-index period.

The risk of discontinuation was calculated for all cohorts and subgroups over time using the Kaplan–Meier method (unadjusted) and Cox proportional hazards models. Variables initially included in the Cox proportional hazards model were sex (male/female), Charlson Comorbidity Index score [9] (≤ 2, 3–5, > 5), and index class (csDMARD, TNF‑α inhibitor, anti-IL‑6 agent, anti-CD80/86 agent, JAK inhibitor). The Cox proportional hazards model was then adjusted for the following additional covariates in the prior csDMARD cohort: analgesic use, glucocorticoid use, chronic kidney disease, anemia, osteoporosis/arthritis, infections, malignancy, diabetes mellitus, dyslipidemia, obesity, venous thromboembolism, depression, liver disease, renal dysfunction or failure (all classed as yes/no), serostatus (seronegative/seropositive/other/unspecified), and index year (2017/2018). The adjusted Cox proportional hazards model was not applied to the prior bDMARD cohort because of the limited sample size. Alluvial diagrams were created to visualize the patient flow between different treatments.

To comply with data protection regulations, sample sizes < 5 could not be reported and, therefore, patient percentages were estimated: since 4 was the highest possible number of patients, the percentages were calculated as 4/*n* (where *n* was the number of patients in the respective group).

## Results

### Patient characteristics of the prior csDMARD and prior bDMARD cohorts

A total of 3858 patients with RA initiating DMARD treatment were identified, of whom 25.7% (*n* = 990) had received prior csDMARD therapy and 9.7% (*n* = 375) prior bDMARD therapy (Table 1). Two patients were excluded during the patient selection process due to implausible treatment combinations (they were prescribed different agents of the same class on the index day). The remaining 2493 patients were DMARD naïve, meaning that they had not received DMARD treatment in the 24-month pre-index period. In both cohorts, there was a greater proportion of female patients (prior csDMARD: 71.9%, prior bDMARD: 74.9%). Mean and median age was 60 years in both the prior csDMARD and prior bDMARD cohorts, with most patients aged between 45 and 64 years; however, a substantial proportion (> 32%) of patients in each cohort were aged 65 years or older.

**Table 1:** Patient and treatment characteristics in the prior csDMARD and prior bDMARD cohorts

	Prior csDMARD cohort *N* = 990	Prior bDMARD cohort *N* = 375
*Female patients, n* (%)	712 (71.9)	281 (74.9)
*Mean (SD) age, years*	60.2 (13.1)	59.8 (11.8)
*Comorbidities, %*		
Injection-site reaction	≤ 0.4^a^	≤ 10.9^a^
Infections	43.6	43.2
Hypersensitivity	10.9	12.5
Gastrointestinal perforation	≤ 0.4^a^	≤ 1.1^a^
Drug poisoning	10.1	10.7
Cardiovascular disorders	61.4	66.7
Bone density/structure disorders	56.5	57.3
Blood dyscrasia	13.7	14.9
Any all-cause hospitalization	45.4	45.6
Venous thromboembolism	4.0	6.9
Thrombosis	5.5	5.3
Pulmonary diseases	23.9	20.3
Other autoimmune diseases	0	0
Metabolic disorders	53.9	51.2
Malignancy	11.2	10.9
Other diseases	55.2	57.3
*Treatment class at index, %*		
csDMARD	23.4	2.7
TNF‑α inhibitor	43.7	13.9
Anti-IL‑6	4.6	3.5
Anti-CD80/86	3.7	3.2
JAK inhibitor	23.7	76.0
*Monotherapy at index, n* (%)	601 (60.7)	291 (77.6)
*Combination therapy at index, n* (%)	389 (39.3)	84 (22.4)
*Glucocorticoid use, %*		
Patients with ≥ 1 glucocorticoid dispensed in the post-index period while on index treatment without glucocorticoid dispensed in the 12-month pre-index period	3.2	4.3
Patients with ≥ 1 glucocorticoid dispensed in the 12-month pre-index period without glucocorticoid dispensed in the post-index period while on index treatment	22.6	19.2
Patients with ≥ 1 glucocorticoid dispensed in the 12-month pre-index period and in the post-index period while on index treatment	63.0	66.1
Patients with ≥ 1 glucocorticoid dispensed in the post-index period while on index treatment	66.3	70.4
Patients with ≥ 1 glucocorticoid dispensed in the 12-month pre-index period	85.7	85.3

*bDMARD* biologic disease-modifying antirheumatic drug, *CD* cluster of differentiation, *csDMARD* conventional synthetic DMARD, *IL* interleukin, *JAK* Janus kinase, *SD* standard deviation, *TNF* tumor necrosis factor

^a^The subgroups comprised samples where *n* < 5, which could not be reported due to data protection regulations; therefore, the percentages could only be estimated

In the pre-index period, specific comorbidities were commonly diagnosed in the two cohorts. Cardiovascular disease was diagnosed in 61.4% and 66.7% of patients, infections were diagnosed in 43.6% and 43.2% of patients, and metabolic disorders were diagnosed in 53.9% and 51.2% of patients in the prior csDMARD and prior bDMARD cohorts, respectively (Table 1**; **Fig. S2). The proportion of patients with diseases of the cardiovascular system at baseline in each treatment group in each cohort is shown in Table S4.

### Treatment characteristics of the prior csDMARD and prior bDMARD cohorts from the pre-index period onwards

More than 60% of patients in the prior csDMARD and prior bDMARD cohorts received prescriptions for glucocorticoids in the pre- and post-index periods. Glucocorticoids were most often prescribed during the 12-month pre-index period, with 85% of patients in both cohorts receiving prescriptions during this time (Table 1). Glucocorticoid use was similar in those receiving monotherapy or combination therapy in both cohorts.

Overall, the most frequently initiated treatments in the prior csDMARD cohort (i.e., the index event) were TNF‑α inhibitors (*n* = 433, 43.7%), followed by JAK inhibitors (*n* = 235, 23.7%) and other csDMARDs (*n* = 232, 23.4%; Table 1). Treatment with other bDMARDs was only initiated in a small proportion of patients in this cohort (anti-IL‑6 agents: *n* = 46, 4.6%; anti-CD80/86 agents: *n* = 37, 3.7%). Patients in the prior bDMARD cohort most frequently initiated treatment with a JAK inhibitor (*n* = 285, 76.0%) or a TNF‑α inhibitor (*n* = 52, 13.9%). The proportion of patients who initiated treatment with other DMARD classes was small (csDMARD: *n* = 10, 2.7%; anti-IL‑6 agents: *n* = 13, 3.5%; anti-CD80/86 agents: *n* = 12, 3.2%). In both cohorts, most patients received monotherapy (prior csDMARD: 60.7%; prior bDMARD: 77.6%) Table 2.

**Table 2:** Patients in the prior csDMARD and prior bDMARD cohorts stratified by monotherapy and combination therapy at index

	Prior csDMARD *n* = 990		Prior bDMARD *n* = 375	
	*n*	%	*n*	%
**Monotherapy at index**				
*Total*	601	60.7	291	77.6
csDMARD	175	17.7	10	2.7
TNF‑α inhibitor	220	22.2	34	9.1
Anti-IL‑1	< 5	–	0	0.0
Anti-IL‑6	30	3.0	8	2.1
Anti-CD20	< 5	–	< 5	–
Anti-CD80/86	13	1.3	8	2.1
JAK inhibitor	157	15.9	228	60.8
**Combination therapy at index**				
*Total*	389	39.3	84	22.4
csDMARD + csDMARD	57	5.8	0	0.0
csDMARD + TNF‑α inhibitor	213	21.5	18	4.8
csDMARD + anti-IL‑1	< 5	–	0	0.0
csDMARD + anti-IL‑6	16	1.6	5	1.3
csDMARD + anti-CD20	0	0.0	0	0.0
csDMARD + anti-CD80/86	24	2.4	< 5	–
csDMARD + JAK inhibitor	78	7.9	57	15.2

*bDMARD* biologic disease-modifying antirheumatic drug, *CD* cluster of differentiation, *csDMARD* conventional synthetic DMARD, *IL* interleukin, *JAK* Janus kinase, *TNF* tumor necrosis factor

As individuals in the prior csDMARD cohort had potentially previously inadequately responded to csDMARD treatment, the csDMARD index agents were assessed in further detail. In the prior csDMARD cohort, leflunomide was the most commonly prescribed index csDMARD monotherapy (39.8%), while leflunomide plus another csDMARD was the most common index csDMARD combination therapy (36.8%; Table 3).

**Table 3:** Index csDMARD treatment received by patients in the prior csDMARD cohort on the index date

	Prior csDMARD	
Index treatment	*n*	%
*csDMARD monotherapy*	176	100.0
Methotrexate	40	22.7
Leflunomide	70	39.8
Hydroxychloroquine sulphate	27	15.3
Sulfasalazine	39	22.2
*csDMARD combination therapy*^a^	57	100.0
Methotrexate + csDMARD^b^	14	24.6
Leflunomide + csDMARD^b^	21	36.8
Hydroxychloroquine sulphate + csDMARD	11	19.3
Sulfasalazine + csDMARD	13	22.8

*csDMARD* conventional synthetic disease-modifying antirheumatic drug

^a^Defined as ≥ 2 csDMARDs

^b^If a patient received methotrexate and leflunomide, they were counted in both categories; therefore, the values total 59 rather than 57, which refers to the distinct number of patients

### Index treatment duration in the prior csDMARD and prior bDMARD cohorts

Median time on index treatment was 215 days in both the prior csDMARD and prior bDMARD cohorts. The mean treatment duration was comparable between the two cohorts (225 days and 228 days, respectively). Within the prior csDMARD cohort, the mean and median treatment duration was higher for patients on monotherapy than for those on combination therapy (mean 240 vs. 202 days; median 275 vs. 177 days; log-rank test *p*-value < 0.01; Table 4). By contrast, in the prior bDMARD cohort, the mean and median treatment duration was higher for combination therapy than monotherapy, although the difference was not statistically significant (mean 233 vs. 226 days; median 226 vs. 214 days; log-rank *p*-value = 0.55; Table 4).

**Table 4:** Discontinuation and persistence duration in the 12-month follow-up period in the prior csDMARD and prior bDMARD cohorts

			Prior csDMARD	Prior bDMARD
Total	Number of patients at risk	*n* (%)	990 (100)	375 (100)
	Number of patients discontinuing	*n* (%)	580 (58.6)	228 (60.8)
	Persistence time (days)	Mean (SD)	224.6 (131.6)	227.6 (126.2)
		Median	214.5	215.0
		Min–max	7.0–365.0	14.0–365.0
Monotherapy	Number of patients at risk	*n* (%)	601 (100)	291 (100)
	Number of patients discontinuing	*n* (%)	321 (53.4)	180 (61.9)
	Persistence time (days)	Mean (SD)	239.6 (130.1)	226.0 (126.1)
		Median	275.0	214.0
		Min–max	11.0–365.0	14.0–365.0
Combination therapy	Number of patients at risk	*n* (%)	389 (100)	84 (100)
	Number of patients discontinuing	*n* (%)	259 (66.6)	48 (57.1)
	Persistence time (days)	Mean (SD)	201.5 (130.6)	233.3 (127.1)
		Median	177.0	225.5
		Min–max	7.0–365.0	28.0–365.0
csDMARD	Number of patients at risk	*n* (%)	232 (100)	10 (100)
	Number of patients discontinuing	*n* (%)	157 (67.7)	6 (60.0)
	Persistence time (days)	Mean (SD)	191.6 (132.5)	211.2 (139.7)
		Median	147.5	180.5
		Min–max	11.0–365.0	50.0–365.0
TNF‑α inhibitor	Number of patients at risk	*n* (%)	433 (100)	52 (100)
	Number of patients discontinuing	*n* (%)	245 (56.6)	37 (71.2)
	Persistence time (days)	Mean (SD)	236.3 (128.2)	209.5 (117.1)
		Median	252.0	180.0
		Min–max	7.0–365.0	14.0–365.0
JAK inhibitor	Number of patients at risk	*n* (%)	235 (100)	285 (100)
	Number of patients discontinuing	*n* (%)	124 (52.8)	170 (59.7)
	Persistence time (days)	Mean (SD)	241.0 (129.4)	231.4 (125.9)
		Median	276.0	232.0
		Min–max	14.0–365.0	28.0–365.0

*bDMARD* biologic disease-modifying antirheumatic drug, *csDMARD* conventional synthetic DMARD, *JAK* Janus kinase, *max* maximum value, *min* minimum value, *SD* standard deviation, *TNF* tumor necrosis factor

**Fig. 2:**
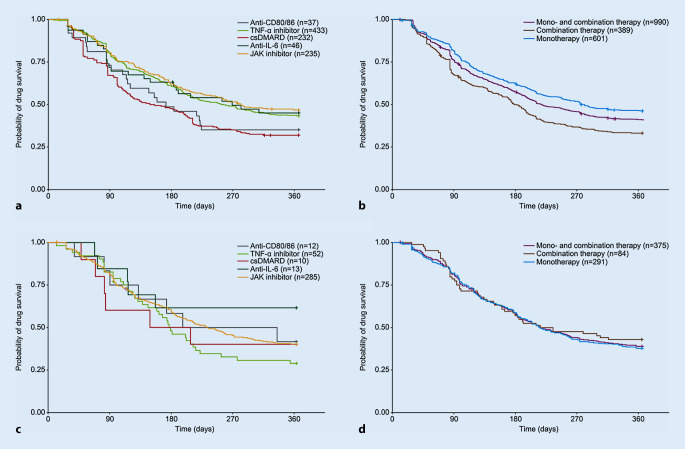
Kaplan–Meier plot of drug persistence for prior csDMARD (**a**,**b**) and prior bDMARD (**c**,**d**) cohorts. In part **c**, the number of patients at risk was very small for anti-CD80/86, csDMARD, and anti-IL‑6 subgroups and, therefore, these data should be interpreted with caution. *bDMARD* biologic disease-modifying antirheumatic drug, *CD* cluster of differentiation, *csDMARD* conventional synthetic DMARD, *IL* interleukin, *JAK* Janus kinase, *TNF* tumor necrosis factor

When considering the treatment classes in the prior csDMARD cohort, individuals remained on treatment the longest when initiating JAK inhibitors (median 276 days), followed by TNF‑α inhibitors (median 252 days) and other csDMARDs (median 148 days; Table 4**; **Fig. 2a). Time on treatment was significantly longer for patients initiating treatment with JAK inhibitors or TNF‑α inhibitors than for those starting on other csDMARDs (log-rank test *p*-value < 0.01 for both comparisons). In the prior csDMARD cohort, the log-rank test indicated a significantly higher probability of persistence for patients on monotherapy than for those on combination therapy (*p* < 0.05; Fig. 2b). In the prior bDMARD cohort, treatment duration was also longest in those initiating JAK inhibitors (median 232 days) and was similar between those initiating csDMARDs and TNF‑α inhibitors (median 181 and 180 days, respectively; Table 4**; **Fig. 2c). However, the log-rank test did not indicate any significant between-group differences (either between treatment classes or therapy types; Fig. 2d), possibly because of the small sample size.

**Fig. 3:**
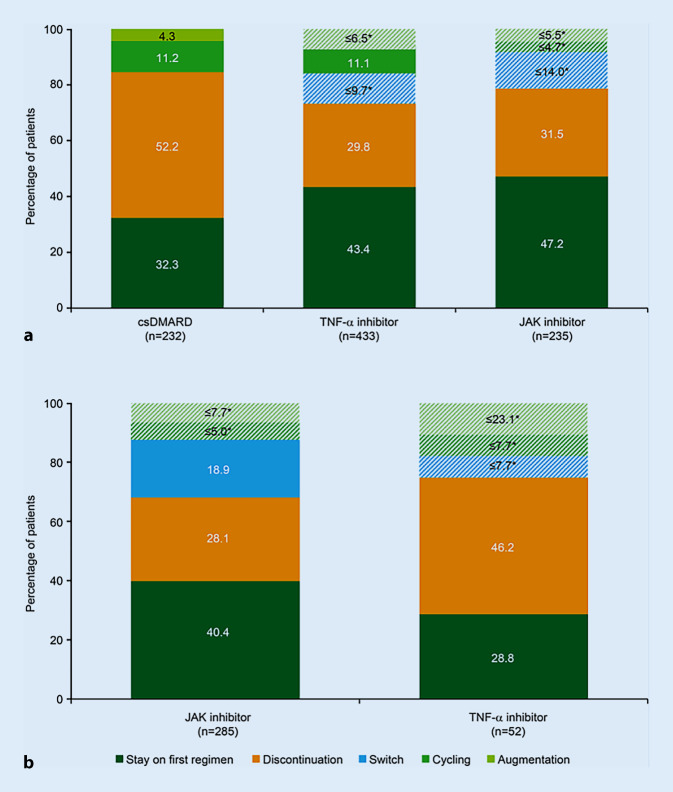
Treatment modifications in the 12-month follow-up in the prior csDMARD (**a**) and prior bDMARD (**b**) cohort. *The subgroups shown as hatched bars comprised samples where *n* < 5, which could not be reported due to data protection regulations; therefore, the percentages could only be estimated. *bDMARD* biologic disease-modifying antirheumatic drug, *csDMARD* conventional synthetic DMARD, *JAK* Janus kinase, *TNF* tumor necrosis factor

In the prior csDMARD cohort, 47.2% of those initiating JAK inhibitors remained on their index treatment class in the 12-month follow-up, compared with 43.4% of patients initiating TNF‑α inhibitors (Fig. 3a). Of those initiating treatment with a different csDMARD, 32.3% remained on their index treatment while 52% discontinued it during the 12-month follow-up period (the remaining patients switched, cycled, or augmented treatment). In both the csDMARD and TNF‑α inhibitor groups, drug cycling was observed in approximately 11% of patients. Switches and therapy augmentations were observed in the TNF‑α inhibitor and JAK inhibitor groups, but owing to the low sample sizes, the rates could only be estimated.

In the prior bDMARD cohort, 40.4% of patients initiating JAK inhibitors remained on this treatment during the 12-month follow-up while only 28.8% of patients initiating TNF‑α inhibitors persisted with this treatment (this difference was not statistically significant, possibly due to the small sample size; Fig. 3b). Of those treated with a JAK inhibitor, 18.9% switched to another drug class. A lower proportion of patients in the TNF‑α inhibitor group switched; however, rates were estimated because of sample size limitations. Drug cycling or augmentation of therapy was observed in up to 12.7% and 30.8% of the JAK inhibitor and TNF‑α inhibitor groups, respectively; however, rates were again estimated owing to sample size restrictions. Low absolute persistence numbers for anti-IL‑6 (21/46 patients) and anti-CD80/86 (13/37 patients) agents in the prior csDMARD cohort, and for TNF‑α inhibitors (15/52 patients), anti-IL‑6 agents (8/13 patients), anti-CD80/86 agents (5/12 patients), and csDMARDs (4/10 patients) in the prior bDMARD cohort limit the validity of the estimates of persistence in these groups.

**Fig. 4:**
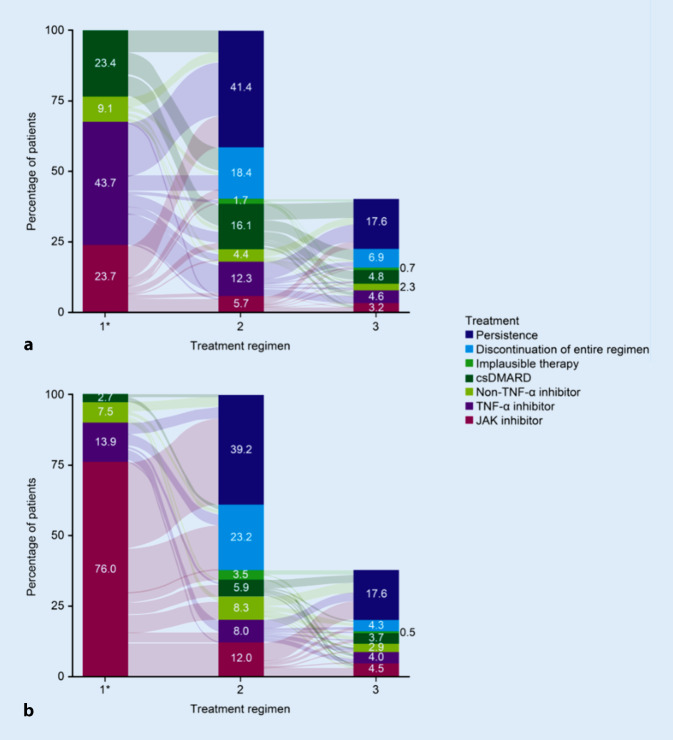
Alluvial diagram of treatment patterns in the prior csDMARD (**a**) and prior bDMARD (**b**) cohorts. Patients classed as changing to the same treatment class (e.g., from a JAK inhibitor to a JAK inhibitor) mostly comprised those who changed their treatment from combination therapy to monotherapy or vice versa. This group also included patients who changed their combination therapy or patients who changed to a different drug from the same class (e.g., from tofacitinib to baricitinib). The bar for the third treatment regimen is shorter than those for the first two regimens because 59.8% of the prior csDMARD cohort and 62.4% of the prior bDMARD cohort were persistent on their index treatment or discontinued their index treatment in the 12-month follow-up and therefore did not receive a third regimen. *Regimen 1 is the index treatment. *bDMARD* biologic disease-modifying antirheumatic drug, *csDMARD* conventional synthetic DMARD, *JAK* Janus kinase, *TNF* tumor necrosis factor

The overall treatment patterns are visualized in Fig. 4.

**Fig. 5:**
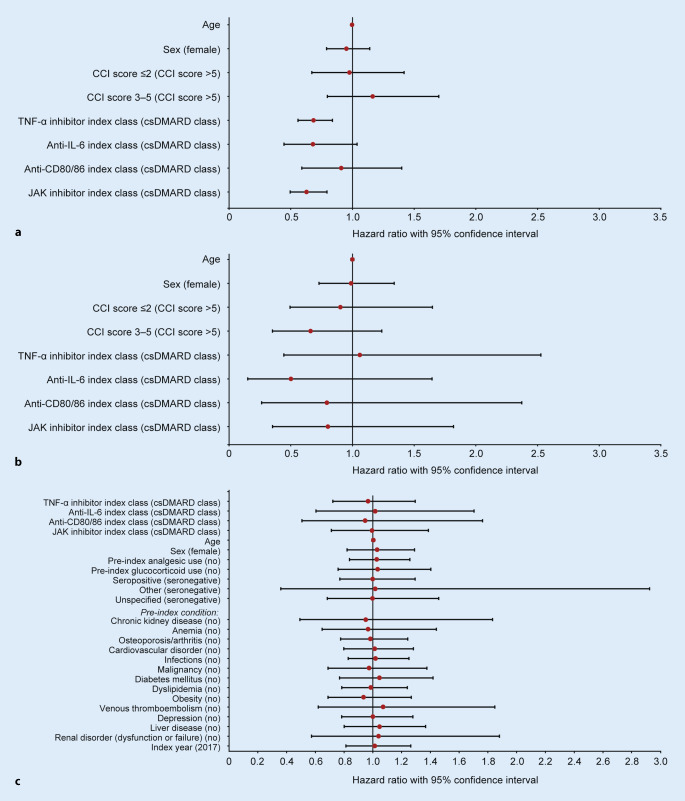
Discontinuation risk in the prior csDMARD (**a**), prior bDMARD (**b**), and prior csDMARD cohort with added variables (**c**). The reference value is stated in brackets for each variable; for treatment comparisons, the csDMARD class was the reference. *bDMARD* biologic disease-modifying antirheumatic drug, *CCI* Charlson Comorbidity Index, *CD* cluster of differentiation, *csDMARD* conventional synthetic DMARD, *IL* interleukin, *JAK* Janus kinase, *TNF* tumor necrosis factor

Cox regression was used to assess whether variables were associated with discontinuation of the index regimen. In the prior csDMARD cohort, the risk of discontinuation decreased if TNF‑α inhibitors or JAK inhibitors were the index treatment class versus the csDMARD reference (hazard ratio [95% confidence interval]: 0.68 [0.56, 0.84] and 0.63 [0.50, 0.79], respectively; Fig. 5a). In the prior bDMARD cohort, none of the regressors had a significant effect on the risk of discontinuation of the index treatment (Fig. 5b), which may be related to the small sample size of this cohort. Additional variables that were assumed to influence persistence, such as pre-index analgesic use, pre-index glucocorticoid use and pre-index conditions, including chronic kidney disease, cardiovascular disease, diabetes mellitus, and depression, were included in an amended Cox regression analysis for the prior csDMARD cohort. None of the additional covariates had a significant effect on the risk of discontinuation (Fig. 5c).

## Discussion

JAK inhibitors have enriched the therapeutic options for patients with RA who inadequately respond to csDMARDs. The extent to which JAK inhibitors have affected treatment patterns since they became available in Germany has not been studied in detail. To our knowledge, this is the first detailed analysis of baseline characteristics and treatment patterns in patients with RA in a real-world setting in Germany that includes JAK inhibitors and focuses on patients who previously received csDMARD or bDMARD therapy.

In the prior csDMARD cohort, the most commonly initiated treatment was a TNF‑α inhibitor; similar proportions of patients initiated treatment with a JAK inhibitor or other csDMARD. In the prior bDMARD cohort, JAK inhibitors were by far the most frequently initiated treatment. In both cohorts, anti-IL‑6 and anti-CD80/86 agents were rarely prescribed, and more patients received monotherapy than combination therapy. This finding is unexpected as the German guidelines advise that bDMARDs are prescribed in combination with MTX where possible. The proportion of patients on monotherapy in both cohorts of our study (61% and 78%) was higher than that reported in the RA Observation of Biologic Therapy (RABBIT), a German registry of patients with RA in which 45.6% of bDMARD-treated patients were receiving no concomitant csDMARD [10]. The higher proportion of patients prescribed monotherapy than combination therapy in our study may be due to poor tolerability experienced with prior MTX/other csDMARD treatment. A history of intolerance would be more relevant in the prior bDMARD cohort (which had the greater proportion of patients on monotherapy), having been escalated from prior csDMARD strategies. Alternatively, due to the selection process, it is possible that combination therapy was underreported in our study.

Glucocorticoid use was common throughout the assessment period. The majority of patients in each cohort (over 85%) received glucocorticoids in the pre-index period, as did over 65% of patients in each cohort in the post-index period while on index treatment. The proportion of patients receiving glucocorticoids during the pre-index phase was slightly higher than that reported in RABBIT (which was included in the JAK-pot study), in which 65% of patients initiating a new DMARD treatment were receiving glucocorticoids at baseline [10]. Data collected from the German national rheumatology database between 1996 and 2016 indicate that the proportion of patients with RA treated with glucocorticoids peaked at approximately 60% in 2001, decreasing to 45% in 2016 [11]. Of note, the German (and the European Alliance of Associations for Rheumatology) guidelines advise that glucocorticoids should be prescribed with initial csDMARD therapy, but reduction to a low dose is recommended within 8 weeks, and the duration of glucocorticoid therapy should be limited to 3–6 months [1, 12].

Of patients in the prior csDMARD cohort, 23% were switched to a different csDMARD or a second csDMARD was added to their treatment regimen as the index event. Furthermore, csDMARD-pretreated patients initiating treatment with JAK inhibitors or TNF‑α inhibitors continued treatment for longer and, as indicated by the log-rank test and Cox regression analysis, were less likely to discontinue their treatment than those remaining on csDMARDs.

This may indicate that another csDMARD therapy in csDMARD-pretreated patients does not result in a lasting response or that it is less well tolerated than other treatments. Consequently, these patients should be monitored even more closely to achieve the treatment goal in accordance with the treat-to-target principle.

However, it is notable that in the prior csDMARD cohort, the majority of patients who received another csDMARD strategy were treated with monotherapy at index (*n* = 175; Table 2). Only a minority of patients (*n* = 57) received combination csDMARD therapy, despite either MTX, SSZ, and HCQ or MTX and LEF being recommended combination treatment options [1]. This finding may partially explain the shorter continuation of a second csDMARD in the prior csDMARD cohort.

Our results indicate that the mean and median treatment duration was longer with monotherapy than with combination therapy within the prior csDMARD cohort, while no significant difference was observed in the prior bDMARD cohort. In contrast, previous German real-world studies have reported longer treatment duration or persistence with combination therapy versus monotherapy. For example, in a recent retrospective study based on claims data, Grellmann et al. found that treatment persistence in patients with RA was longest in those receiving anti-TNF therapy in combination with a csDMARD, with approximately 50% of patients remaining on treatment for 270 days, compared with approximately 30% of those on anti-TNF therapy alone [13]. In addition, based on data from RABBIT, Zink et al. found that drug continuation rates were higher for etanercept or infliximab when combined with MTX or another DMARD than for either agent alone [14]. Our contrasting finding may reflect a switch from combination therapy to monotherapy during the follow-up period once patients have achieved remission or low disease activity, as persistence was defined conservatively. Moreover, the group of patients on combination therapy might reflect a population with higher disease activity and treatment resistance, leading to an earlier switch to other drugs.

Comorbidities such as cardiovascular disease, infections, and metabolic disorders were common in the prior csDMARD and prior bDMARD cohorts, which is in line with previous reports [13, 15–17]. When baseline cardiovascular diseases were compared across index classes, a comparatively high proportion of patients treated with anti-CD80/86 agents had hypertension or ischemic heart disease. These comorbidities were reported in a higher proportion of patients treated with JAK inhibitors than TNF‑α inhibitors, indicating a greater baseline cardiovascular risk in the former group. The finding of longer treatment duration with JAK inhibitors compared with TNF inhibitors in the current study, despite less favorable cardiovascular risk profile in the JAK inhibitor cohort, is of note in light of the recently published Oral Surveillance study which failed to show non-inferiority of tofacitinib compared to TNF inhibitors in a cardiovascular risk population [18]. In the Oral Surveillance study, the mean treatment duration was similar in the tofacitinib 5 mg and 10 mg groups and the TNF inhibitor group (41.1 and 38.5 months and 40.2 months, respectively); therefore, the cardiovascular risk appeared to have no obvious impact on treatment persistence [18].

There are a number of limitations to the current analyses, including the low patient numbers, which may explain the lack of significant differences for some of the regressions performed. Another limitation was the applied hierarchy used to determine the index treatment (JAK inhibitor > bDMARD > csDMARD), which resulted in a shift towards JAK inhibitors in terms of patient numbers. The hierarchy could have also influenced the switching and augmentation patterns within the prior csDMARD and prior bDMARD groups in the 12-month follow-up, which was limited to the index years 2017 and 2018. For example, csDMARD-treated patients who switched to JAK inhibitors in 2017 were included in the JAK inhibitor cohort and did not qualify as “csDMARD switchers.” This means that the numbers of switches and augmentations in particular could be underestimated, which, in turn, could have led to overestimation of the duration of treatment (“time on treatment”) in the csDMARD and bDMARD groups. More specifically, individuals who initiated treatment with a csDMARD and switched to a JAK inhibitor in 2017/2018 were not included in the csDMARD cohort by default, and so forth. This could have overestimated treatment duration in csDMARD/bDMARD-treated patients in comparison with those receiving JAK inhibitor treatment. Nevertheless, patients initiating treatment with JAK inhibitors or TNF‑α inhibitors remained on treatment for longer than did those on csDMARDs. A further limitation was that patients in the prior csDMARD and prior bDMARD cohorts had had at least one previous csDMARD or bDMARD prescribed, respectively, in the 24-month pre-index period. As this was based on prescriptions rather than clinical factors, patients experiencing inadequate response to treatment (failing to achieve treatment targets) could not be differentiated from those with an intolerance to treatment. As the pre-index period was restricted to 2 years, earlier data were not captured and, therefore, the number of patients reported may be underestimated. In addition, at the time the analysis was performed, tofacitinib and baricitinib were the only JAK inhibitors available in Germany. Since that time, upadacitinib and filgotinib have also been approved for treatment of RA in Europe. Finally, claims data are primarily collected for accounting purposes; thus, clinical parameters such as disease severity grades, duration of disease, prognostic factors, and laboratory results were not captured. In addition, the numbers of previous treatment changes were unavailable. Time on treatment was based on prescription data, including the date of medication collection and the recommended dose as per the package leaflet. Therefore, it is not known if a physician prescribed a different dosing pattern or non-standard dose, or whether the patient took their medication as prescribed (e.g., they may have stopped treatment before taking the whole pack).

## Conclusion

This analysis provides detailed information regarding treatment patterns and baseline characteristics in patients with RA in a real-world setting in Germany since the introduction of JAK inhibitors. Such data are important to understand how patients are treated in clinical practice and to identify whether improvements can be made to optimize patient care and outcomes. We found that TNF‑α inhibitors and JAK inhibitors were the most commonly prescribed DMARD class in the prior csDMARD and prior bDMARD cohorts, respectively. Only a minority of patients received another csDMARD strategy in the prior csDMARD group, and this mainly as monotherapy. In both the prior csDMARD and prior bDMARD cohorts, more patients received monotherapy than combination therapy, which was unexpected, as the German guidelines advocate combination treatments when escalating treatment from initial csDMARD therapy. Time on treatment was significantly longer for patients initiating treatment with JAK inhibitors or TNF‑α inhibitors than for those starting on other csDMARDs in the prior csDMARD cohort. However, there was no statistically significant difference in drug persistence between JAK inhibitors and TNF‑α inhibitors in this cohort. In the prior bDMARD cohort, approximately 40% of patients initiating treatment with JAK inhibitors continued treatment during the 12-month follow-up, compared with approximately 29% of those initiating treatment with TNF‑α inhibitors.

### Supplementary Information

**Table S1** ICD-10-GM codes used to identify rheumatoid arthritis (inclusion criterion)**, Table S2 **OPS and ATC codes to identify DMARD (inclusion criterion)**, Table S3 **ICD-10-GM codes to identify pregnancy or autoimmune diseases other than rheumatoid arthritis (exclusion criterion)**, Table S4 **Diseases of the circulatory system at baseline occurring in ≥ 5% of a treatment group**, Fig. S1 **Study design**, Fig. S2 **Prevalence of specific comorbidities in the 12-month pre-index period

## Declarations

Anonymized statutory health insurance data were used for this study. The use of such data is not subject to ethics committee approval in Germany. *Consent for publication:* not applicable.
