# Supplementary material for: Real-world treatment persistence in patients with rheumatoid arthritis initiating DMARDs in Germany—a health insurance claims data analysis
Source: Z Rheumatol. 2023 Feb 9;82(9):739–53. doi: 10.1007/s00393-023-01323-8 (PMC10627963; doi:10.1007/s00393-023-01323-8)
Supplement: Supplementary file 1 — Table S1 ICD-10-GM codes used to identify rheumatoid arthritis (inclusion criterion), Table S2 OPS and ATC codes to identify DMARD (inclusion criterion), Table S3 ICD-10-GM codes to identify pregnancy or autoimmune diseases other than rheumatoid arthritis (exclusion criterion), Table S4 Diseases of the circulatory system at baseline occurring in ≥ 5% of a treatment group, Fig. S1 Study design, Fig. S2 Prevalence of specific comorbidities in the 12-month pre-index period [file 393_2023_1323_MOESM1_ESM.pdf]

Supplementary information

Table S1 ICD-10-GM codes used to identify rheumatoid arthritis (inclusion criterion)*

Code	Code_Type	Group 1	Group 2
M05	ICD-10-GM	Seropositive chronic polyarthritis	RA
M050	ICD-10-GM	Felty's syndrome	RA
M051	ICD-10-GM	Pulmonary manifestation of seropositive chronic polyarthritis	RA
M052	ICD-10-GM	Vasculitis in seropositive chronic polyarthritis	RA
M053	ICD-10-GM	Seropositive chronic polyarthritis with involvement of other organs and organ systems	RA
M058	ICD-10-GM	Other sero-positive chronic polyarthritis	RA
M059	ICD-10-GM	Seropositive chronic polyarthritis, unspecified	RA
M060	ICD-10-GM	Seronegative chronic polyarthritis	RA
M068	ICD-10-GM	Other specified chronic rheumatoid arthritis	RA
M069	ICD-10-GM	Chronic polyarthritis, unspecified	RA

*Patients were required to have at least two RA diagnoses within 12 months (i.e., 4 quarters), with at least one of them coded by a certified rheumatologist. Rheumatologists were identified:

a) in the outpatient setting using the two digits “31” representing the specialty “Rheumatologie/Innere Medizin” (rheumatology/internal medicine) of the lifelong physician number which is given to every physician by the statutory health insurance in Germany (Lebenslange Arztnummer – LANR) or
b) in the inpatient setting by a rheumatological department stay.

ICD-10-GM, International Statistical Classification of Diseases and Related Health Problems, 10th revision, German Modification

Table S2 OPS and ATC codes to identify DMARD (inclusion criterion)

Group	Class	Agent	OPS	ATC
csDMARD	--	Hydroxychloroquine sulfate	--	P01BA02
csDMARD	--	Leflunomide	--	L04AA13
csDMARD	--	Methotrexate	--	L04AX03 & M01CX01
csDMARD	--	Sulfasalazine	--	M01CX02
bDMARD	Anti-CD80/86	Abatacept	6-003.s0	L04AA24
bDMARD	TNF-α inhibitor	Adalimumab	6-001.d2 & 6-001.d3	L04AB04
bDMARD	TNF-α inhibitor	Certolizumab pegol	6-005.7	L04AB05
bDMARD	TNF-α inhibitor	Etanercept	6-002.b1	L04AB01
bDMARD	TNF-α inhibitor	Golimumab	6-005.2	L04AB06
bDMARD	TNF-α inhibitor	Infliximab	6-001.e	L04AB02
bDMARD	AntiCD20	Rituximab	6-001.h8	L01XC02
bDMARD	Anti-IL-6	Sarilumab	6-00a.g	L04AC14
bDMARD	Anti-IL-6	Tocilizumab	6-005.m	L04AC07
tsDMARD	JAKi	Baricitinib	--	L04AA37
tsDMARD	JAKi	Tofacitinib	--	L04AA29

ATC, Anatomical Therapeutic Classification; bDMARD, biologic disease-modifying anti-rheumatic drug; csDMARD, conventional synthetic disease-modifying anti-rheumatic drug; DMARD, disease-modifying anti-rheumatic drug; OPS, Operationen- und Prozedurenschlüssel

Table S3 ICD-10-GM codes to identify pregnancy or autoimmune diseases other than rheumatoid arthritis (exclusion criterion)

Code	Code_Type	Group 1	Group 2	Group 3
H20	ICD-10-GM	Iridocyclitis	non-infectious uveitis	non-infectious uveitis
H200	ICD-10-GM	Acute and subacute iridocyclitits	non-infectious uveitis	non-infectious uveitis
H201	ICD-10-GM	Chronic iridocyclitis	non-infectious uveitis	non-infectious uveitis
H202	ICD-10-GM	Phakogenic iridocyclitis	non-infectious uveitis	non-infectious uveitis
H208	ICD-10-GM	Other iridocyclitis	non-infectious uveitis	non-infectious uveitis
H209	ICD-10-GM	Iridocyclitis, not specified	non-infectious uveitis	non-infectious uveitis
H302	ICD-10-GM	Posterior cyclitis	non-infectious uveitis	non-infectious uveitis
H308	ICD-10-GM	Other chorioretinitis (incl. Harada's disease)	non-infectious uveitis	non-infectious uveitis
H441	ICD-10-GM	Other endophthalmitis	non-infectious uveitis	non-infectious uveitis
K50	ICD-10-GM	Crohn's disease [regional enteritis] [Crohn's disease]	Crohn's disease	Autoimmune diseases
K51	ICD-10-GM	Ulcerative colitis	Ulcerative colitis	Autoimmune diseases
K603	ICD-10-GM	Anal fistula	Anal fistula	Autoimmune diseases
L40	ICD-10-GM	Psoriasis	Psoriasis	Autoimmune diseases
L400	ICD-10-GM	Psoriasis vulgaris	Psoriasis	Autoimmune diseases
L401	ICD-10-GM	Generalized psoriasis pustulosa	Psoriasis	Autoimmune diseases
L402	ICD-10-GM	Acrodermatitis continua suppurativa	Psoriasis	Autoimmune diseases
L403	ICD-10-GM	Psoriasis pustulosa palmoplantaris	Psoriasis	Autoimmune diseases
L404	ICD-10-GM	Psoriasis guttata	Psoriasis	Autoimmune diseases
L405	ICD-10-GM	Psoriatic arthropathy	Psoriatic arthritis	Autoimmune diseases
L408	ICD-10-GM	Other psoriasis	Psoriasis	Autoimmune diseases
L409	ICD-10-GM	Psoriasis, unspecified	Psoriasis	Autoimmune diseases
L732	ICD-10-GM	Hidradenitis suppurativa	Hidradenitis suppurativa	Hidradenitis suppurativa
L93	ICD-10-GM	Lupus erythematosus	Lupus	Autoimmune diseases
M070	ICD-10-GM	Distal interphalangeal psoriatic arthritis	Psoriatic arthritis	Autoimmune diseases
M071	ICD-10-GM	Arthritis mutilans	Psoriatic arthritis	Autoimmune diseases
M072	ICD-10-GM	Psoriatica spondylitis	Psoriatic arthritis	Autoimmune diseases
M073	ICD-10-GM	Other psoriatic arthritis	Psoriatic arthritis	Autoimmune diseases
M08	ICD-10-GM	Juvenile arthritis	Juvenile arthritis	Autoimmune diseases
M090	ICD-10-GM	Juvenile arthritis in psoriasis	Psoriatic arthritis	Autoimmune diseases

M321	ICD-10-GM	Systemic lupus erythematosus w organ or system involvement	Lupus	Autoimmune diseases
M328	ICD-10-GM	Other forms of systemic lupus erythematosus	Lupus	Autoimmune diseases
M329	ICD-10-GM	Systemic lupus erythematosus, unspecified	Lupus	Autoimmune diseases
M45	ICD-10-GM	Ankylosing spondylitis	Ankylosing spondylitis	Autoimmune diseases
M450	ICD-10-GM	Ankylosing spondylitis	Ankylosing spondylitis	Autoimmune diseases
O09	ICD-10-GM	duration of pregnancy	Pregnancy	Pregnancy
O10	ICD-10-GM	Pre-existing hypertension that complicates pregnancy, birth and puerperium	Pregnancy	Pregnancy
O11	ICD-10-GM	Chronic hypertension with grafted preeclampsia	Pregnancy	Pregnancy
O12	ICD-10-GM	Gestational edema and gestational proteinuria [pregnancy-induced] without hypertension	Pregnancy	Pregnancy
O13	ICD-10-GM	Gestational hypertension [pregnancy-induced hypertension]	Pregnancy	Pregnancy
O14	ICD-10-GM	pre-eclampsia	Pregnancy	Pregnancy
O15	ICD-10-GM	eclampsia	Pregnancy	Pregnancy
O16	ICD-10-GM	Unspecified hypertension of the mother	Pregnancy	Pregnancy
O20	ICD-10-GM	Bleeding in early pregnancy	Pregnancy	Pregnancy
O21	ICD-10-GM	Excessive vomiting during pregnancy	Pregnancy	Pregnancy
O22	ICD-10-GM	Venous diseases and hemorrhoids as complications in pregnancy	Pregnancy	Pregnancy
O23	ICD-10-GM	Infections of the genitourinary tract in pregnancy	Pregnancy	Pregnancy
O24	ICD-10-GM	Diabetes mellitus in pregnancy	Pregnancy	Pregnancy
O25	ICD-10-GM	Malnutrition in pregnancy	Pregnancy	Pregnancy
O26	ICD-10-GM	Care of the mother in other conditions, which are mainly associated with the pregnancy	Pregnancy	Pregnancy
O28	ICD-10-GM	Abnormal findings in the screening of the mother for prenatal diagnosis	Pregnancy	Pregnancy
O29	ICD-10-GM	Complications of anesthesia during pregnancy	Pregnancy	Pregnancy
O30	ICD-10-GM	Multiple pregnancy	Pregnancy	Pregnancy
O31	ICD-10-GM	Complications specific to a multiple pregnancy	Pregnancy	Pregnancy
O32	ICD-10-GM	Care of the mother in case of established or suspected abnormal position and adjustment of the fetus	Pregnancy	Pregnancy
O33	ICD-10-GM	Care of the mother in case of established or assumed mismatch between fetus and pelvis	Pregnancy	Pregnancy

O34	ICD-10-GM	Care of the mother in case of established or suspected anomaly of the pelvic organs	Pregnancy	Pregnancy
O35	ICD-10-GM	Care of the mother in case of established or suspected anomaly or damage to the fetus	Pregnancy	Pregnancy
O36	ICD-10-GM	Care of the mother for other identified or suspected complications in the fetus	Pregnancy	Pregnancy
O40	ICD-10-GM	polyhydramnios	Pregnancy	Pregnancy
O41	ICD-10-GM	Other changes of the amniotic fluid and the membranes	Pregnancy	Pregnancy
O42	ICD-10-GM	Premature rupture of membranes	Pregnancy	Pregnancy
O43	ICD-10-GM	Pathological states of the placenta	Pregnancy	Pregnancy
O44	ICD-10-GM	Placenta previa	Pregnancy	Pregnancy
O45	ICD-10-GM	Premature placental abruption [Abruptio placentae]	Pregnancy	Pregnancy
O46	ICD-10-GM	Prepartial bleeding, not elsewhere classified	Pregnancy	Pregnancy
O47	ICD-10-GM	Frustrane Contractions [useless labor pain]	Pregnancy	Pregnancy
O48	ICD-10-GM	Prolonged pregnancy	Pregnancy	Pregnancy
O60	ICD-10-GM	Premature labor and delivery	Pregnancy	Pregnancy
O61	ICD-10-GM	Failed induction of labor	Pregnancy	Pregnancy
O62	ICD-10-GM	Abnormal labor activity	Pregnancy	Pregnancy
O63	ICD-10-GM	Protracted birth	Pregnancy	Pregnancy
O64	ICD-10-GM	Birth obstacle due to position, posture and adjustment abnormalities of the fetus	Pregnancy	Pregnancy
O65	ICD-10-GM	Obstacle due to abnormality of the maternal pelvis	Pregnancy	Pregnancy
O66	ICD-10-GM	Other obstacle during childbirth	Pregnancy	Pregnancy
O67	ICD-10-GM	Complications of labor and delivery due to intrapartum hemorrhage, not elsewhere classified	Pregnancy	Pregnancy
O68	ICD-10-GM	Complications of labor and delivery due to fetal distress [fetal distress]	Pregnancy	Pregnancy
O69	ICD-10-GM	Complications during labor and delivery due to umbilical cord complications	Pregnancy	Pregnancy
O70	ICD-10-GM	Dam rupture during delivery	Pregnancy	Pregnancy
O71	ICD-10-GM	Other injuries during childbirth	Pregnancy	Pregnancy
O72	ICD-10-GM	Postpartum hemorrhage	Pregnancy	Pregnancy
O73	ICD-10-GM	Retention of the placenta and the membranes without bleeding	Pregnancy	Pregnancy
O74	ICD-10-GM	Complications of anesthesia during labor and childbirth	Pregnancy	Pregnancy

O75	ICD-10-GM	Other complications of labor and delivery, not elsewhere classified	Pregnancy	Pregnancy
O80	ICD-10-GM	Spontaneous delivery of a singleton	Pregnancy	Pregnancy
O81	ICD-10-GM	Birth of a singleton by forceps or vacuum extraction	Pregnancy	Pregnancy
O82	ICD-10-GM	Birth of a singleton with caesarean section	Pregnancy	Pregnancy
O85	ICD-10-GM	puerperal fever	Pregnancy	Pregnancy
O86	ICD-10-GM	Other puerperal infections	Pregnancy	Pregnancy
O87	ICD-10-GM	Venous diseases and hemorrhoids as complications in the puerperium	Pregnancy	Pregnancy
O88	ICD-10-GM	Embolism during the gestation period	Pregnancy	Pregnancy
O89	ICD-10-GM	Complications of anesthesia in the puerperium	Pregnancy	Pregnancy
O90	ICD-10-GM	Puerperal complications, not elsewhere classified	Pregnancy	Pregnancy
O91	ICD-10-GM	Infections of the breast [mammary gland] in connection with gestation	Pregnancy	Pregnancy
O92	ICD-10-GM	Other diseases of the breast [mammary gland] associated with gestation and lactation disorders	Pregnancy	Pregnancy
O94	ICD-10-GM	Consequences of complications during pregnancy, childbirth and puerperium	Pregnancy	Pregnancy
O95	ICD-10-GM	Death during gestation period unspecified cause	Pregnancy	Pregnancy
O96	ICD-10-GM	Death as a result of any gestational cause after more than 42 days to less than one year after delivery	Pregnancy	Pregnancy
O97	ICD-10-GM	Death due to the causes of gestation	Pregnancy	Pregnancy
O98	ICD-10-GM	Infectious and parasitic diseases of the mother, which can be classified elsewhere, but which complicate pregnancy, childbirth and the puerperium	Pregnancy	Pregnancy
O99	ICD-10-GM	Other maternal diseases that can be classified elsewhere, but which complicate pregnancy, childbirth and the puerperium	Pregnancy	Pregnancy

ICD-10-GM, International Statistical Classification of Diseases and Related Health Problems, 10th revision, German Modification

Table S4 Diseases of the circulatory system at baseline occurring in ≥5% of a treatment group

	Prior csDMARD										Prior bDMARD									
	**csDMARD class n=232**		**TNF-α inhibitor index class n=433**		**Anti-IL-6 index class n=46**		**Anti-CD80/86 index class n=37**		**JAK inhibitor index class n=235**		**csDMARD class n=10**		**TNF-α inhibitor index class n=52**		**Anti-IL-6 index class n=13**		**Anti-CD80/86 index class n=12**		**JAK inhibitor index class n=285**	
	**n**	**%**	**n**	**%**	**n**	**%**	**n**	**%**	**n**	**%**	**n**	**%**	**n**	**%**	**n**	**%**	**n**	**%**	**n**	**%**
Essential (primary) hypertension	120	51.7	217	50.1	28	60.9	28	75.7	130	55.3	7	70.0	22	42.3	9	69.2	10	83.3	168	58.9
Varicose veins of lower extremities	47	20.3	60	13.9	0	0.0	8	21.6	41	17.4	0	0.0	6	11.5	0	0.0	0	0.0	36	12.6
Chronic ischaemic heart disease	30	12.9	37	8.5	0	0.0	12	32.4	33	14.0	0	0.0	0	0.0	0	0.0	0	0.0	42	14.7
Atherosclerosis	20	8.6	29	6.7	0	0.0	0	0.0	21	8.9	0	0.0	6	11.5	0	0.0	0	0.0	25	8.8
Other cardiac arrhythmias	16	6.9	35	8.1	0	0.0	0	0.0	21	8.9	0	0.0	7	13.5	0	0.0	0	0.0	22	7.7
Hypertensive heart disease	16	6.9	24	5.5	0	0.0	6	16.2	18	7.7	0	0.0	6	11.5	0	0.0	0	0.0	24	8.4
Heart failure	22	9.5	24	5.5	0	0.0	7	18.9	24	10.2	0	0.0	0	0.0	0	0.0	0	0.0	25	8.8
Atrial fibrillation and flutter	12	5.2	22	5.1	0	0.0	6	16.2	19	8.1	0	0.0	0	0.0	0	0.0	0	0.0	19	6.7
Other disorders of veins	19	8.2	29	6.7	0	0.0	0	0.0	16	6.8	0	0.0	0	0.0	0	0.0	0	0.0	22	7.7
Nonrheumatic mitral valve disorders	10	4.3	19	4.4	0	0.0	0	0.0	11	4.7	0	0.0	0	0.0	0	0.0	0	0.0	18	6.3
Other cerebrovascular diseases	14	6.0	11	2.5	0	0.0	0	0.0	8	3.4	0	0.0	0	0.0	0	0.0	0	0.0	13	4.6
Occlusion and stenosis of precerebral arteries, not resulting in cerebral infarction	11	4.7	17	3.9	0	0.0	0	0.0	9	3.8	0	0.0	0	0.0	0	0.0	0	0.0	15	5.3

Other peripheral vascular diseases	10	4.3	23	5.3	0	0.0	0	0.0	12	5.1	0	0.0	0	0.0	0	0.0	0	0.0	12	4.2
Phlebitis and thrombophlebitis	10	4.3	11	2.5	0	0.0	0	0.0	7	3.0	0	0.0	0	0.0	0	0.0	0	0.0	15	5.3

bDMARD, biologic disease-modifying anti-rheumatic drug; CD, cluster of differentiation; csDMARD, conventional synthetic disease-modifying anti-rheumatic drug; IL, interleukin;JAK, Janus kinase; TNF, tumour necrosis factor

Fig. S1 Study design

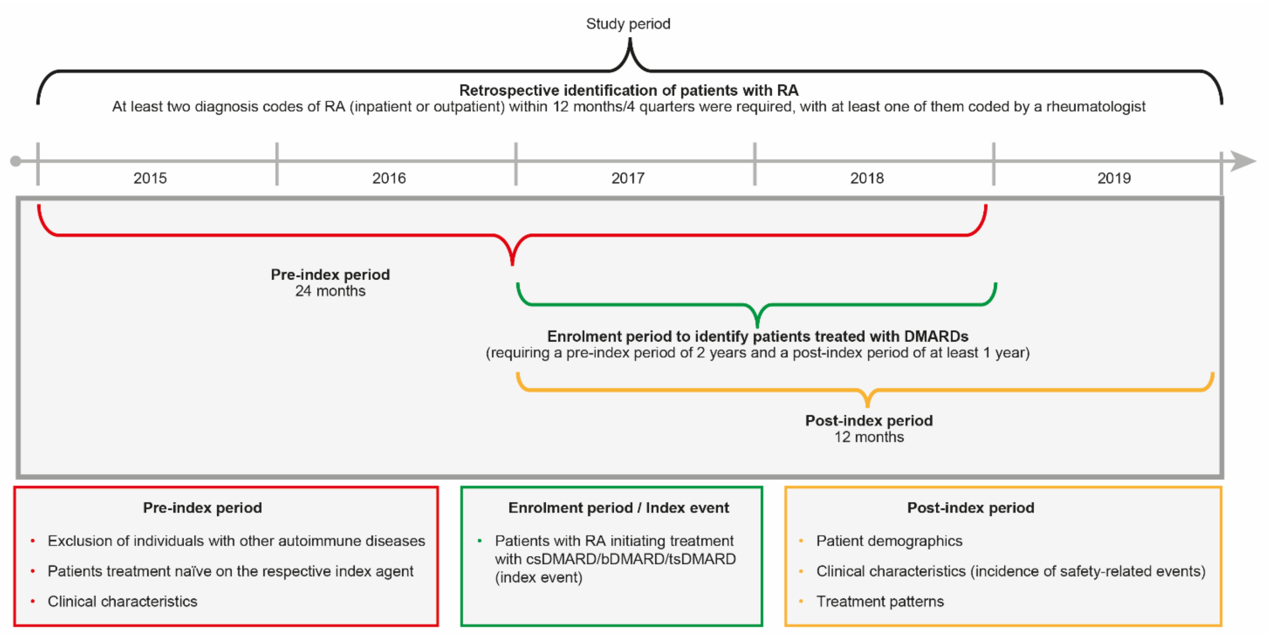

bDMARD, biologic disease-modifying anti-rheumatic drug; csDMARD, conventional synthetic disease-modifying anti-rheumatic drug; RA, rheumatoid arthritis; tsDMARD, targeted synthetic disease-modifying anti-rheumatic drug

Fig. S2 Prevalence of specific comorbidities in the 12-month pre-index period

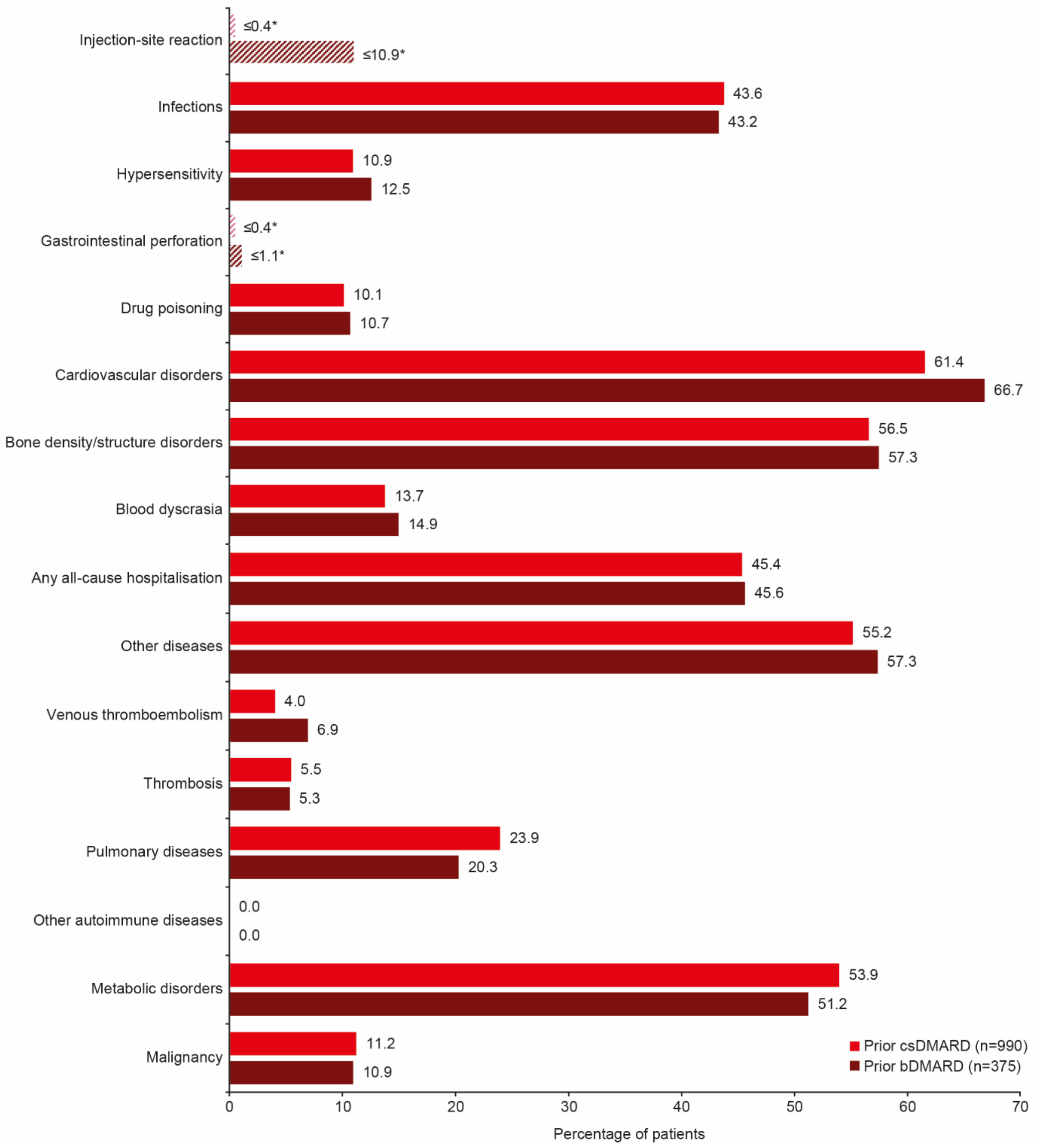

*The subgroups shown as hatched bars comprised samples where n<5, which could not be reported due to data protection regulations; therefore, the percentages could only be estimated.

bDMARD, biologic disease-modifying anti-rheumatic drug; csDMARD, conventional synthetic disease-modifying anti-rheumatic drug; IR, inadequate response
